# Enquête sur la contrefaçon de quelques anti-infectieux administrés per os commercialisés dans la ville de Lubumbashi

**DOI:** 10.11604/pamj.2015.22.318.7302

**Published:** 2015-12-02

**Authors:** Pierrot Mwamba Tshilumba, Salvius Bakari Amuri, Elie Rongorongo Kaghowa, Danny Mukeba Mbikay, Alex Bokanya Impele, Pierre Duez, Jean Baptiste Kalonji Ndoumba

**Affiliations:** 1 Laboratoire de Pharmacie Galénique, Analyse des Médicament & Contrôle Qualité, Faculté des Sciences Pharmaceutiques, Université de Lubumbashi, Commune Kampemba, Lubumbashi, RD Congo; 2 Laboratoire de Chimie Thérapeutique et Pharmacognosie, Faculté de Médecine et de Pharmacie, Université de Mons (UMONS), Bâtiment Mendeleiev, Av. Maistriau, Belgique; 3 Laboratoire de Pharmacognosie, Faculté des Sciences Pharmaceutiques Université de Lubumbashi, Commune Kampemba, Lubumbashi, RD Congo

**Keywords:** Médicaments, contrefaçon, anti-infectieux, Lubumbashi, RD Congo, Medicines, counterfeit, antimicrobial, Lubumbashi, DR Congo

## Abstract

**Introduction:**

La production, la vente et la consommation des médicaments contrefaits sont de sérieux problèmes qui pèsent sur la Santé Publique particulièrement des pays en développement et pour lesquels il existe peu d'informations dans certains pays. Cette étude a été menée en vue de contribuer à la détermination de la prévalence des médicaments contrefaits, particulièrement celle des anti-infectieux consommés dans la ville de Lubumbashi en République Démocratique du Congo.

**Méthodes:**

L’étude a visé des médicaments anti-infectieux administrés per os commercialisés dans la ville de Lubumbashi. L'inspection visuelle attentive de l'emballage, l'interprétation de l’étiquetage et l'observation attentive du produit ont servi de paramètres d’études. Les échantillons ont été acquis par achat auprès des fournisseurs licites et illicites.

**Résultats:**

Cinq molécules: ampicilline, amoxycilline, ciprofloxacine, mebendazole et metronidazole ont été colletées. Sur 60 échantillons rassemblés: 31,7% se sont avérés contrefaits. L'ampicilline et le mebendazole sont les produits les plus contrefaits dans cette étude avec 26,3%, suivi de metronidazole avec 21,05%. 78,9% des médicaments contrefaits proviennent du secteur informel. La provenance de 47,4% d'anti-infectieux contrefaits est l'Inde, suivi de la Chine avec 26,3%.

**Conclusion:**

Cette étude a montré la circulation des médicaments contrefaits dans la ville de Lubumbashi à un taux non négligeable. Une étude des caractéristiques physico-chimiques et de l'activité biologiques permettra d’évaluer l'impact de ces médicaments dans la prise en charge des infections.

## Introduction

Un médicament contrefait est un médicament qui est délibérément et frauduleusement étiqueté pour tromper sur son identité et/ou sur son origine [1]. La contrefaçon peut concerner les médicaments de marque déposée comme les produits génériques. On trouve également dans les contrefaçons de médicaments des produits avec les principes actifs corrects, erronés, sans principe actif, à des doses trop faibles ou trop fortes, ou sous des conditionnements falsifiés [1]. La contrefaçon pharmaceutique est un fléau sanitaire, responsable de plusieurs cas de morbidité et de mortalités [2, 3]. La prise des médicaments contrefaits augmente les risques d’échec du traitement des diverses affections qui frappent la population, elle peut aussi favoriser plusieurs intoxications médicamenteuses [3–5]. Sur le plan économique, le quart des médicaments en circulation commerciale dans le monde serait contrefait, leur valeur atteignait 75 milliards dollars américains en 2010 [4–6]. La contrefaçon pharmaceutique constitue également un réel manque à gagner pour les recettes de l'Etat et pour les industriels [6]. Des études montrent que la circulation des médicaments contrefaits concerne surtout les pays en développement où l'application de la réglementation pharmaceutique y manque de rigueur, la présence de marché illicite y connait une expansion et les contrôles y sont peu rigoureux [2, 3]. En Afrique, l'Organisation Mondiale de la Santé (OMS) estime jusqu’ à 25%, la présence des médicaments contrefaits ou non conformes [2, 7]. En République Démocratique du Congo, particulièrement dans la ville de Lubumbashi, les statistiques précises sur la prévalence des médicaments contrefaits ne sont pas disponibles. Aucune étude de grande ampleur et de méthodologie rigoureuse n'a été réalisée jusqu'ici. Pourtant, l'abondance des produits pharmaceutiques vendus dans les marchés de rue et le nombre croissant des échecs thérapeutiques supposeraient la circulation, toute aussi importante, des médicaments contrefaits [8]. Les maladies infectieuses et leur prise en charge constituent un problème majeur de Santé Publique. Leur prise en charge est compliquée par les phénomènes de résistance [3, 9]. En Afrique, surtout dans les régions tropicales comme la R. D. Congo, les infections représentent la majorité des causes de morbi-mortalité [10, 11]. Le besoin croissant des anti-infectieux fait d'eux les médicaments susceptibles d’être contrefaits. Ils représentent 28% de contrefaçon dans le monde [6, 9, 12]. C'est en considérant toutes les préoccupations susmentionnées que cette étude est menée en vue de contribuer à la détermination de la prévalence des médicaments contrefaits particulièrement celle des anti-infectieux commercialisés dans la ville de Lubumbashi en RD Congo.

## Méthodes

### Milieu

Sur le plan géographique cette étude a concerné les médicaments obtenus à Lubumbashi, capitale du Katanga, province du sud-est de la République démocratique du Congo. La descente sur terrain a été effectuée dans les sept communes (Lubumbashi, Kampemba, Kamalondo, Kenya, Katuba, Ruashi et Annexe) de la ville de Lubumbashi. L'enquête a été orientée vers les structures pharmaceutiques formelles (officine ouverte au public, pharmacie interne des hôpitaux, établissement de vente en gros) et informelles (Etalages des médicaments de marché, marché des rues pharmaceutiques).

### Matériel

La sélection des produits a été réalisée suivant les critères ci-après: faire partie des produits les plus utilisés, être sur la liste nationale des médicaments essentiels, faire partie des produits les plus susceptibles d’être contrefait [7]. Les principes actifs concernés par cette étude sont l'amoxycilline (capsule dosée à 500 mg et 250 mg), l'ampicilline (capsule dosée à 500 mg et 250 mg), ciprofloxacine (comprimé dosé à 500 mg), mébendazole (comprimé dosé à 100 mg et 500 mg), métronidazole (comprimé dosé à 500 mg et 250 mg) et les images d’étiquetage ont été prises par un appareil photo numérique de marque Sony corps model no dsc-w120.

### Méthode

La contrefaçon d'amoxycilline, d'ampicilline, de ciprofloxacine, de mébendazole et de métronidazole a été mise en évidence par l'inspection visuelle attentive de l'emballage, l'interprétation de l’étiquetage et l'observation attentive du produit. Cette méthode simple mais efficace présente l'avantage de la détection rapide de la contrefaçon, car selon l'OMS, l'identification d'un médicament potentiellement contrefait passe en premier lieu par l'inspection visuelle attentive du produit ainsi que par l’étiquetage [2, 4, 5, 7]. La collecte des échantillons s'est déroulée du 30 Juillet 2012 au 25 Août 2012. La sélection des points de collecte a été réalisée de façon aléatoire dans les marchés ou dans la rue ainsi que dans les différentes structures pharmaceutiques licites. Les échantillons provenant d'un même point de vente et portant un étiquetage indiquant les mêmes mentions: dénomination commune internationale, nom de marque, nom et adresse du fabricant ou distributeur, numéro de l'autorisation de mise sur le marché, dosage, numéro de lot, date de fabrication et de péremption, numéro de lot, emplacement des mentions, ont été considérés comme un seul et même échantillon [2].

## Résultats

**Figure 1:**
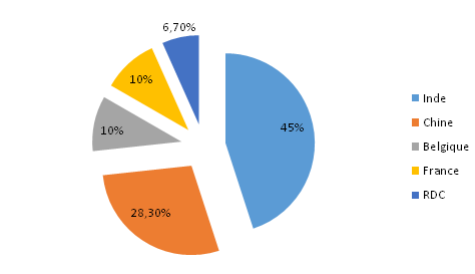
Répartition des échantillons collectés selon les pays de provenance

Sur 60 échantillons, l'ampicilline est le produit le plus représenté avec 23,3% suivi de mébendazole (21,7%), d'amoxycilline (20%) et respectivement 18,3% et 16,7% pour la ciprofloxacine et le métronidazole. 41,7% d’échantillons collectés dans cette étude proviennent des officines ouvertes aux publics, suivi des établissement de marché de rues pharmaceutiques (35%), des établissements de vente en gros (13,3%) puis des pharmacies internes des hôpitaux (10%). Sur 60 échantillons rassemblés dans cette étude, 19 soit 31,7% se sont avérés contrefaits (Tableau 1). Il est aussi à noter que 45% d’échantillons collectés proviennent d'Inde (Figure 1).

**Tableau 1:** Répartition des échantillons contrefaits et non contrefaits selon leur site de collecte

Principe actif	Nombre d’échantillon collectés	Echantillons collectés dans les structures licites		Echantillons collectés dans les structures illicites	
		Ech. non contrefait	Ech. contrefait	Ech. non contrefait	Ech. contrefait
Ampicilline	14	8	2	1	3
Amoxycilline	12	8	-	1	3
Ciprofloxacine	11	6	-	3	2
Mébendazole	13	7	1	1	4
Métronidazole	10	6	-	-	4

## Discussion

**Figure 2:**
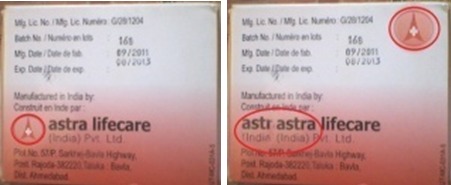
Exemple de contrefaçon d'ampicilline produite par Astra lifecare

**Figure 3:**
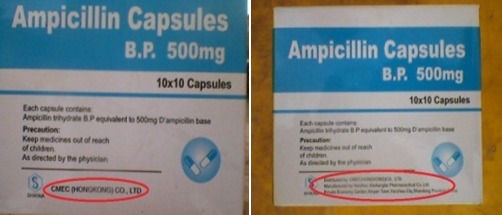
Exemple de contrefaçon d'ampicilline produite par Shikina

**Figure 4:**
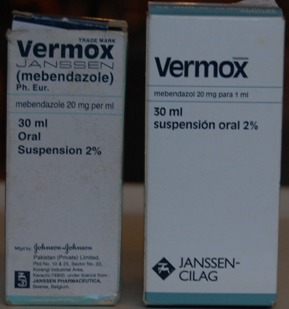
Exemple de contrefaçon de mébendazole produit par Janssen

**Figure 5:**
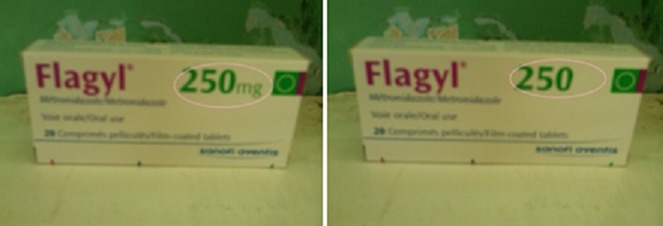
Exemple de contrefaçon de métronidazole produit par Sanofi

**Figure 6:**
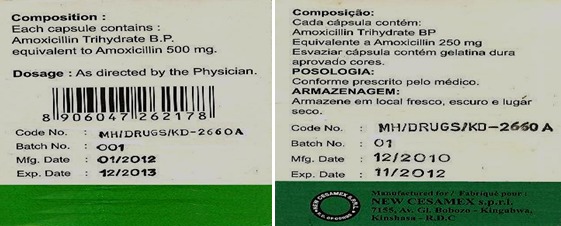
Exemple de contrefaçon d'amoxicilline produite par Newcesamex

**Figure 7:**
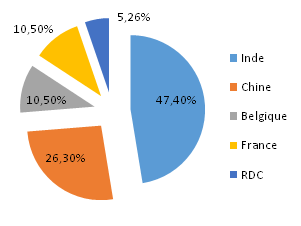
Répartition des échantillons contrefaits selon les pays d'origine

Dans cette étude, 31,7% (n = 60) des anti-infectieux collectés ont été suspectés de contrefaçon selon les critères de l'OMS. Cette observation est différente de celle faite par Diarietou (2001) qui a trouvé que 14,8% (n= 54) échantillons d'anti-infectieux sélectionnés dans son étude (ampicilline, amoxycilline, cotrimoxazole et tétracycline), étaient contrefaits. Cette étude montre également un taux élevé d'antibiotiques contrefaits comparativement aux résultats de Delepierre et al (2012), qui stipulent que la contrefaçon des anti-infectieux représente 28% dans le monde [12]. Tous les cinq principes actifs sélectionnés dans cette étude sont touchés par la contrefaçon: l'ampicilline (Figure 2, Figure 3) et le mébendazole (Figure 4) occupent la première place avec 26,3% de contrefaçon, suivi de métronidazole (Figure 5) avec 21,05%, 15,8% pour l'amoxycilline (Figure 6) et 10,52% pour la ciprofloxacine. Ceci se rapproche de constat fait en Côte d'Ivoire par Legris (2005) qui a trouvé que le mébendazole était le principe actif le plus contrefait dans la classe d'antiparasitaire (14%) par contre 2% de cas de contrefaçon avaient concerné l'ampicilline [2]. Diarietou (2001) a trouvé dans son étude que l'ampicilline était le produit le plus contrefait (30,8%) parmi les médicaments anti-infectieux sélectionnés [13]. Par ailleurs, Il est à noter également que 78,9% des anti-infectieux suspecté de contrefaçon collectés dans cette étude, proviennent des établissements de marché de rues pharmaceutiques. Cette constatation corrobore les données avancées par plusieurs études selon lesquelles: la présence importante du marché illicite est un facteur potentiel favorisant la circulation des médicaments contrefaits [2, 8, 14]. Ceci reste inquiétant car la population lushoise a recours massivement à ce marché. D'une part, 47,4% des anti-infectieux suspectés de contrefaçon sont originaires d'Inde, suivi de la Chine (26,3%) (Figure 7). Cette constatation rejoint l'affirmation de l'OMS qui a publié que plus de 35% des médicaments contrefaits sont produits en Inde [15], pour leur part Delepierre et al. (2012) ont trouvé que 78% des anti-infectieux falsifiés détectés dans leur étude étaient originaires de l'Asie [12]. De ce qui précède, il est observé que la contrefaçon des anti-infectieux en République Démocratique du Congo, plus précisément dans la ville de Lubumbashi est un réel problème sanitaire dans le marché lushois par la forte présence de marché illicite commercialisant les médicaments, ceci reste préoccupant car la population lushoise a recourt à ces établissement pharmaceutiques illicites.

## Conclusion

Cette étude a porté sur l'enquête de la contrefaçon des anti-infectieux commercialisés à Lubumbashi, deuxième ville, en République Démocratique du Congo. L'enquête s'est orientée vers les structures licites et illicites. Il ressort de cette étude que l'inspection de l’étiquetage de 60 échantillons d'anti-infectieux obtenus dans différents sites, 31,7% ont été suspecté de contrefaçon. Cette contrefaçon est largement dominée par le marché illicite étant donné que 78,9% des médicaments suspectés de contrefaçon proviennent du secteur informe l.47,4% des anti-infectieux suspectés de contrefaçon sont originaires d'Inde, suivi de la Chine (26,3%). Il est possible que la lutte contre la présence des marchés illicites de médicaments puisse diminuer l'afflux des médicaments contrefaits dans cette ville. Il est en outre indispensable qu'un contrôle renforcé au niveau des frontières soit permanant afin de réduire la prévalence des médicaments contrefaits dans la ville de Lubumbashi. Dans l'avenir une étude plus poussée et élargie portant sur le contrôle des propriétés physico chimiques, des caractéristiques biopharmaceutiques des échantillons déclarés comme contrefaits devra être entreprise dans la ville de Lubumbashi et ses environs.
